# Robust Adaptive HCS MPPT Algorithm-Based Wind Generation System Using Model Reference Adaptive Control

**DOI:** 10.3390/s21155187

**Published:** 2021-07-30

**Authors:** Ziyad A. Alrowaili, Mustafa M. Ali, Abdelraheem Youssef, Hossam H. H. Mousa, Ahmed S. Ali, Gamal T. Abdel-Jaber, Mohammed Ezzeldien, Fatma Gami

**Affiliations:** 1 Department of Physics, College of Science, Jouf University, Sakaka P.O. Box 2014, Saudi Arabia; zalrowaili@ju.edu.sa (Z.A.A.); Meabas@ju.edu.sa (M.E.); 2 Department of Mechanical Engineering, South Valley University, Qena 83523, Egypt; gtag2000@yahoo.com; 3 Department of Electrical Engineering, South Valley University, Qena 83523, Egypt; A.yousaf@eng.svu.edu.eg (A.Y.); H.Herzallah@eng.svu.edu.eg (H.H.H.M.); 4 Department of Mechanical Engineering, Assiut University, Assiut 71511, Egypt; ahmadsaad01@yahoo.com; 5 Metallurgy & Material Science Tests (MMST) Lab, Department of Physics, Faculty of Science, South Valley University, Qena 83523, Egypt; 6 Department of Physics, College of Science and Arts, Jouf University, Al-Qurayat Branch P.O. Box 756, Saudi Arabia; fsgamie@ju.edu.sa; 7 Physics Department, South Valley University, Qena 83523, Egypt

**Keywords:** HCS, MRAC, PID, dynamic step size, WECS

## Abstract

To treat the stochastic wind nature, it is required to attain all available power from the wind energy conversion system (WECS). Therefore, several maximum power point tracking (MPPT) techniques are utilized. Among them, hill-climbing search (HCS) techniques are widely implemented owing to their various features. Regarding current HCS techniques, the rotor speed is mainly perturbed using predefined constants or objective functions, which makes the selection of step sizes a multifaceted task. These limitations are directly reflected in the overall dynamic WECS performance such as tracking speed, power fluctuations, and system efficiency. To deal with the challenges of the existing HCS techniques, this paper proposes a new adaptive HCS (AD-HCS) technique with self-adjustable step size using model reference adaptive control (MRAC) based on the PID controller. Firstly, the mechanical power fluctuations are detected, then the MRAC continuously optimizes the PID gains so as to generate an appropriate dynamic step size until harvesting the maximum power point (MPP) under the optimal tracking conditions. Looking specifically at the simulation results, the proposed AD-HCS technique exhibits low oscillations around the MPP and a small settling time. Moreover, WECS efficiency is increased by 5% and 2% compared to the conventional and recent HCS techniques, respectively. Finally, the studied system is confirmed over a 1.5 MW, gird-tied, double-fed induction generator (DFIG) WECS using MATLAB/Simulink.

## 1. Introduction

Pressing ecological issues, particularly concerning global warming, have drawn attention to renewable energy sources (RESs) [1,2]. In view of RESs, wind energy is the most extensive renewable source. However, it still demands several high-tech enhancements [3,4]. Therefore, applying new control techniques in wind energy conversion systems (WECSs) plays a key role in the manufacture, guaranteeing their cost-effectiveness and high performance efficiency [5].

**Figure 1:**
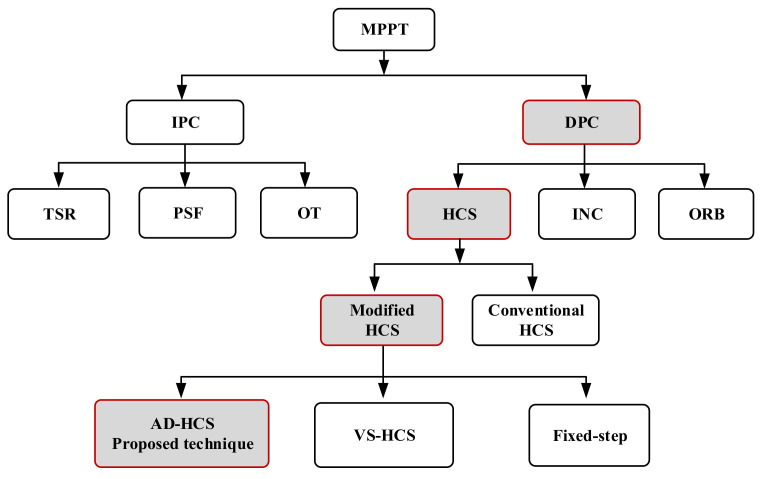
MPPT strategies classifications.

To extract all available wind power, several researchers have developed different maximum power point tracking (MPPT) techniques for regulating the generated mechanical power from the wind turbine (WT) under changeable climate environments. In recent literature [6,7], the MPPT algorithms are clustered into the direct power controller (DPC) and indirect power controller (IPC), as shown in Figure 1.

In view of DPC algorithms, the power variations according to a prestored WT curve are observed, and the maximum power point (MPP) is tracked. However, the WT parameters have not been required. On the other hand, the IPC depends on precise knowledge of the WT [8,9]. IPC algorithms involve the tip speed ratio (TSR) [10,11], the power signal feedback (PSF) [12,13], and the optimal torque (OT) [14,15]. Although TSR is a simple and easy method, it requires precise information about the wind speed, which necessitates several distributed mechanical sensors around the swept area with 5–10% accuracy. Although the PSF and OT techniques do not require mechanical sensors, they still need the exact parameters of the WT [16].

“Otherwise, the hill-climbing search (HCS) or the perturb and observe (P&O) [17,18], optimum relation-based (ORB) [19], and incremental conductance (INC) MPPT techniques are considered as DPC techniques” [20,21]. Looking deeply at the HCS technique, it is applied extensively for the assembly of all available power under different wind speeds. Besides, the HCS technique has many features, such as simplicity and flexibility. Moreover, the HCS does not necessitate distributed mechanical sensors for measuring wind speed. In view of the HCS technique, its tracking strategy depends on perturbing the rotor speed with an appropriate step size then analyzing the variation in the extracted power until the slope of the power–speed curve turns to zero [22]. In order to regulate the tracking speed, the HCS technique may use either fixed step size or variable step size. The traditional HCS technique depends on a fixed step size, which can be small or large. If the small step size is applied, the small steady-state oscillations around the MPP are observed, but the tracking response of the rotor speed is slow, which decreases the average output power. In contrast, the large step size has a rapid response with large power oscillations in the steady-state period, which decreases the WT efficiency [22,23,24,25]. Hence, the enhancement of the HCS technique necessitates fine-tuning of the step size and perturbation direction to accomplish MPP tracking requirements at overall wind speeds [26]. To increase the HCS operation capability and exterminate the drawbacks, various adaptive and variable step size techniques have been implemented in [27,28,29,30]. In [31], the authors have proposed an adaptive HCS to decrease the mechanical transmission stress between the WT and the generator. An adaptive duty-cycle HCS strategy dependent on gradient approximation has been suggested in [32]. However, with the extreme variations in wind speed, the MPP can swiftly change. Therefore, the mechanical stress may not be significantly decreased. A fast strategy that utilizes two tracking stages might fail during the rapid fluctuations of wind speed [33]. Despite using anemometers for wind-speed measurement, [22] used large forward step size and small reverse step size, which successfully accelerated the tracking speed under fast wind fluctuations. Based on a speed-sensorless tracking process with adaptive perturbation step size, the suggested algorithm in [2] can effectively offer the solution to recent tracking drawbacks, in which the rectifier input voltage and current are utilized. Similarly, in [34], the previous strategy with minor modifications is applied. However, it involves many assumptions that decrease overall machine efficiency. To further follow the developments of the HCS technique, a literature review of several papers is summarized in Table 1. 

To overcome the limitations of recent HCS techniques as investigated in [6], this paper proposes a new adaptive HCS (AD-HCS) technique with a dynamic perturbation step size for a grid-tied double-fed induction generator (DFIG)-based WECS. Furthermore, the main objectives of the proposed technique can be summarized as follows:

- The suggested algorithm has the ability to regulate rotor speed under different operating conditions by using the model reference adaptive control (MRAC) based on PID.
- Dependent on observing the mechanical power fluctuations, the MRAC continuously tunes the PID gains in order to generate a suitable dynamic step size (Ȿ) until tracking the MPP under the optimal tracking circumstances.
- Regarding the recent HCS techniques, the step size basically depends on predefined constants or objective functions, which makes the choosing of step sizes a complex task. However, the proposed technique is considered as a self-modulation of step size without prior knowledge of system parameters and memory requirements.
- As a result of applying the proposed technique, the optimum power extraction with high efficiency, low oscillations and fast response compared to existing HCS techniques is attained.

The framework of this paper is as follows: Section 2 presents a brief description of a WECS-based DFIG. Section 3 discusses the conventional HCS algorithm. Section 4 illustrates the MRAC strategy. The proposed HCS method is presented in Section 5. Section 6 presents the simulation results of the suggested technique. Finally, the conclusions are presented in Section 7.

## 2. Studied Variable-Speed WECS Modeling

**Figure 2:**
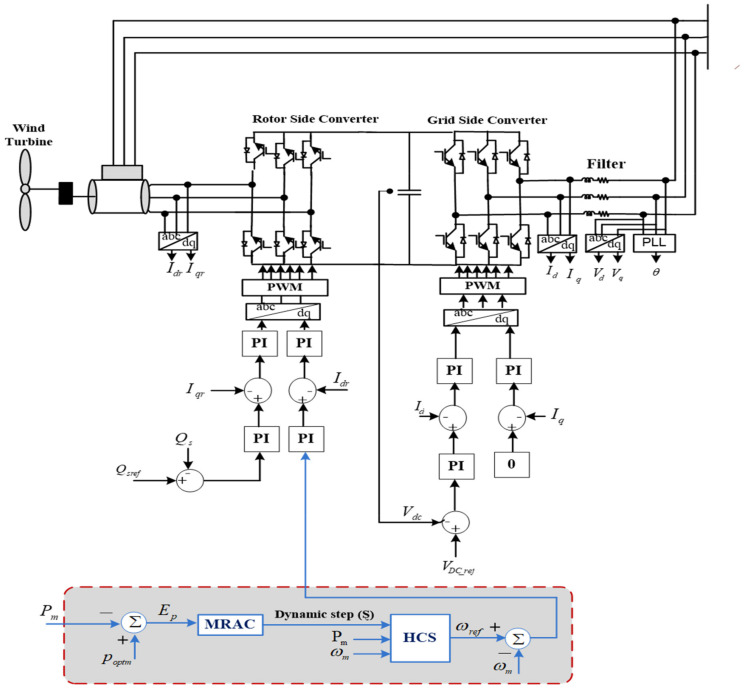
Studied system.

Figure 2 explains the generic arrangement of the 1.5 MW, grid-tied, variable-speed WECS-based DFIG in which the rotor-side converter (RSC) and the grid-side converter (GSC) are operated through the back-to-back (BTB) converter, which is linked via a DC link [48,49].

### 2.1. Aerodynamic Model

The dynamic wind energy, which is addressed by a mass, m, streaming at wind speed, $v_{w}$, in the x-direction, is expressed as follows:

(1) $$U=\frac{1}{2}{\mathrm{mv}}_{w}{}^{2}=\frac{1}{2}(\rho \mathrm{Ax})v_{w}{}^{2}$$

Here, U is the kinetic energy (joule), ρ denotes air density (kg/m^3^), A indicates swept rotor area ($m^{2})$, and x indicates the air thickness (m). Wind power, $P_{w}$, can be obtained using

(2) $$P_{w}=\frac{\mathrm{dU}}{\mathrm{dt}}=\frac{1}{2}{\;\rho \mathrm{Av}}_{w}{}^{2}\frac{\mathrm{dx}}{\mathrm{dt}}=\frac{1}{2}{\;\rho \mathrm{Av}}_{w}{}^{3}$$

Here, $A={\pi R}^{2}$, where R expresses blade radius (m).

### 2.2. Wind Turbine Model

**Figure 3:**
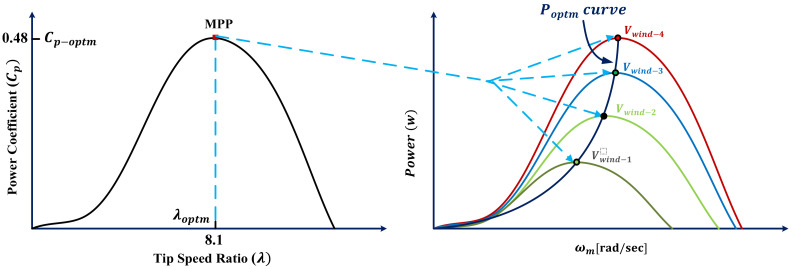
Power characteristic curve [6].

The harvested mechanical power, $P_{m}$, from the WT is attained as follows [27,50]:

(3) $$P_{m}=\frac{1}{2}{\pi \rho R}^{2}C_{p}\left(\lambda ,\beta \right)v_{w}{}^{3}$$

where $C_{p}\;$ denotes power coefficient, λ indicates TSR and β is pitch angle. In this study, the pitch angle is assumed to be zero, and the TSR and the $C_{p}$ are formulated as

(4) $$\lambda =\frac{\omega_{m}R}{v_{w}}$$

(5) $$C_{p}\left(\lambda ,\beta \right)=C_{1}\left(\frac{C_{2}}{\lambda_{i}}-C_{3}\beta -C_{4}\;\right)e^{\frac{-{\;C}_{5}}{\lambda_{i}}}+C_{6}\lambda_{i}$$

(6) $$\lambda_{i}={\left[\frac{1}{\left(\lambda +C_{7}.\beta \right)}-\frac{C_{8}}{\left(\beta^{3}+1\right)}\right]}^{-1}$$

where $\omega_{m}$ refers to the rotor speed. By looking deeply in Equations (4)–(6), it is clear that $C_{p}$ is based on the rotor speed only. For achieving the optimal power from WT, the $C_{p}$ should be 0.48 and the λ should be 8.1. The characteristic power–speed curve for various wind speeds is depicted in Figure 3.

### 2.3. Shaft System Modeling

To simulate the WECS, a single mass model with a lumped inertia constant $H_{m}$ is used, as given in [51]:

(7) $$H_{m}=H_{t}+H_{g}$$

where $H_{t}$ and $H_{g}$ denotes the inertia constants of the WT and generator, respectively. Subsequently, the dynamic system is expressed as follows:

(8) $$\frac{d}{\mathrm{dt}}\omega_{m}=\frac{1}{2H_{m}}\;\left(T_{m}-T_{e}-{D\omega }_{m}\right)$$

$T_{m}$ is the mechanical torque presented by $T_{m}=p_{m}/\omega_{m}$, D indicates lumped damping factor and $T_{e}$ is the electromagnetic torque.

### 2.4. DFIG Model

**Figure 4:**
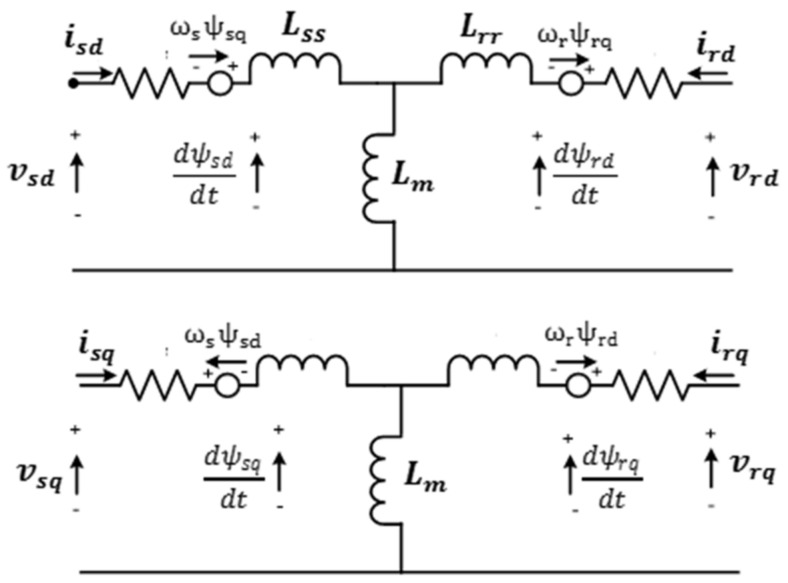
Equivalent circuit of d−q axis of DFIG rotor voltages.

As shown in Figure 4, the stator and the rotor voltages are presented as investigated in [52,53], expressed as

(9) $$\{\begin{array}{c} v_{\mathrm{sd}}=R_{s}i_{\mathrm{Sd}}+\frac{d}{\mathrm{dt}}\psi_{\mathrm{sd}}-\omega_{s}\psi_{\mathrm{sq}}\\ v_{\mathrm{sq}}=R_{s}i_{\mathrm{Sq}}+\frac{d}{\mathrm{dt}}\psi_{\mathrm{sq}}+\omega_{s}\psi_{\mathrm{sd}} \end{array}$$

(10) $$\{\begin{array}{c} v_{\mathrm{rd}}=R_{r}i_{\mathrm{rd}}+\frac{d}{\mathrm{dt}}\psi_{\mathrm{rd}}-\omega_{r}\psi_{\mathrm{rq}}\\ v_{\mathrm{rq}}=R_{r}i_{\mathrm{rq}}+\frac{d}{\mathrm{dt}}\psi_{\mathrm{rq}}+\omega_{r}\psi_{\mathrm{rd}} \end{array}$$

where $i_{\mathrm{Sd}-q\;}and$ $i_{\mathrm{rd}-q}\;\mathrm{are}\;\mathrm{the}\;d-q\;\mathrm{stator}\;\mathrm{and}\;\mathrm{rotor}\;\mathrm{currents},{\;R}_{r}\;and{\;R}_{s}$ denote rotor and stator resistances and $\omega_{r}\;and{\;\omega }_{s}$ are the electrical rotor and stator voltage speed.

Mathematical expressions for the d–q stator and rotor fluxes ($\psi_{\mathrm{sd}-q},\psi_{\mathrm{rd}-q}$) are specified as

(11) $$\{\{\begin{array}{c} \psi_{\mathrm{sd}}=L_{\mathrm{ss}}i_{\mathrm{sd}}+L_{m}i_{\mathrm{rd}}\\ \psi_{\mathrm{sq}}=L_{\mathrm{ss}}i_{\mathrm{sq}}+L_{m}i_{\mathrm{rq}} \end{array}$$

(12) $$\{\begin{array}{c} \psi_{\mathrm{rd}}=L_{m}i_{\mathrm{sd}}+L_{\mathrm{rr}}i_{\mathrm{rd}}\\ \;\psi_{\mathrm{rq}}=L_{m}i_{\mathrm{sq}}+L_{\mathrm{rr}}i_{\mathrm{rq}} \end{array}$$

where L_ss_, L_rr_ and L_m_ denote stator, rotor and magnetizing inductances, respectively, by observing that $L_{ss}=L_{m}+L_{s}$ and $L_{rr}=L_{m}+L_{r}$.

Now, the electromagnetic torque $T_{\mathrm{em}}\;$ expression can be obtained by the following equation:

(13) $$T_{em}=\frac{3}{2}P\frac{L_{m}}{L_{\mathrm{ss}}}\left(\psi_{\mathrm{sq}}i_{\mathrm{rd}}-\psi_{\mathrm{sd}}i_{\mathrm{rq}}\right)$$

where P is the number of poles. 

### 2.5. Rotor-Side Converter

For an accurate simulation of the RSC to track the optimal power under swift variation in wind speed, there are two control loops, named the speed and current control loops, as depicted in Figure 2. The speed control loop is utilized to extract the reference generator speed in the different climate conditions, which are regulated via the MPPT algorithm. Alternatively, the current control loop is utilized for controlling the generator current to specify the operating switching pulses. The d–q stator flux voltages ($v_{\mathrm{sd}}$, $v_{\mathrm{sq}}$) are formulated by [52]

(14) $$\{\begin{array}{c} v_{\mathrm{sd}}=-\omega_{s}\psi_{\mathrm{sq}}\\ v_{\mathrm{sq}}=\omega_{s}\psi_{\mathrm{sd}} \end{array}$$

By using $\psi_{sd}$≈0, the stator active and reactive powers ($P_{s}$,${\;Q}_{s}$) are clarified by

(15) $$\{\begin{array}{c} P_{s}=-\frac{3}{2}\;\frac{L_{m}}{L_{\mathrm{ss}}}\left[V_{\mathrm{sd}}{\;i}_{\mathrm{rd}}\;\right]\;\\ Q_{s}=\frac{3}{2}\;\frac{v_{\mathrm{sd}}{}^{2}}{\omega_{s}L_{\mathrm{ss}}}+\frac{3}{2}v_{\mathrm{sd}}\frac{L_{m}}{L_{\mathrm{ss}}}i_{\mathrm{rq}} \end{array}$$

Here, the d–q rotor voltages ($v_{\mathrm{rd}\;},v_{\mathrm{rq}}$) are stated as follows:

(16) $$\{\begin{array}{c} v_{\mathrm{rd}\;}=R_{r}i_{\mathrm{rd}}+\sigma L_{\mathrm{rr}}\frac{{\mathrm{di}}_{\mathrm{rd}}}{\mathrm{dt}}-\left(\sigma \omega_{r}L_{\mathrm{rr}}i_{\mathrm{rq}}+\omega_{r}\frac{L_{m}}{L_{\mathrm{ss}}}\psi_{\mathrm{sq}}\right)\\ v_{\mathrm{rq}}=R_{r}i_{\mathrm{rq}}+\sigma L_{\mathrm{rr}}\frac{{\mathrm{di}}_{\mathrm{rq}}}{\mathrm{dt}}+\sigma \omega_{r}L_{\mathrm{rr}}i_{\mathrm{rd}}\; \end{array}$$

Hence, $\sigma =(L_{rr}-\frac{L_{m}{}^{2}}{L_{ss}})$. 

Finally, the electromagnetic torque will become

(17) $$T_{\mathrm{em}}=\frac{3}{2}\;P\;\frac{L_{m}}{L_{\mathrm{ss}}}{\;\psi }_{\mathrm{sq}}i_{\mathrm{rd}}$$

### 2.6. Grid Side Converter

The DC-link voltage ($V_{Dc}$) of the GSC is maintained at a fixed value for the complete flow of the active power between both converter sides, besides controlling the exchanged reactive power from the utility grid. The GSC shown in Figure 2 involves dual cascaded controller loops. Therefore, it is expressed by the following equations [52]:

(18) $$\{\begin{array}{c} v_{\mathrm{fd}}=-\left(R_{f}i_{\mathrm{dg}}+L_{f}\frac{{\mathrm{di}}_{\mathrm{dg}}}{\mathrm{dt}}\right)+v_{\mathrm{sd}}+\omega_{s}L_{f}i_{\mathrm{qg}}\\ v_{\mathrm{fq}}=-\left(R_{f}i_{\mathrm{qg}}+L_{f}\frac{{\mathrm{di}}_{\mathrm{qg}}}{\mathrm{dt}}\right)-\omega_{s}L_{f}i_{\mathrm{dg}}\; \end{array}$$

(19) $$\{\begin{array}{c} P_{g}=\frac{3}{2}\left(v_{\mathrm{sd}}i_{\mathrm{dg}}+v_{\mathrm{sq}}i_{\mathrm{qg}}\right)=\frac{3}{2}v_{\mathrm{sd}}i_{\mathrm{dg}\;}\\ Q_{g}=\frac{3}{2}\left(v_{\mathrm{sq}}i_{\mathrm{dg}}-v_{\mathrm{sd}}i_{\mathrm{qg}}\right)=-\frac{3}{2}v_{\mathrm{sd}}i_{\mathrm{qg}} \end{array}$$

where ${\;R}_{f}$ and $L_{f}\;$ are the filter resistance and inductance, respectively. $v_{fd}\;and\;v_{fq}\;$ denote d–q axis of output voltages, $i_{dg}\;and\;i_{qg}$ express d–q axis of grid currents and $v_{sd}$ is d-axis of grid voltage, where $v_{sq}\;assumed\;zero.$ $P_{g}$ and $Q_{g}$ are the output active and reactive powers.

## 3. Conventional HCS Technique 

**Figure 5:**
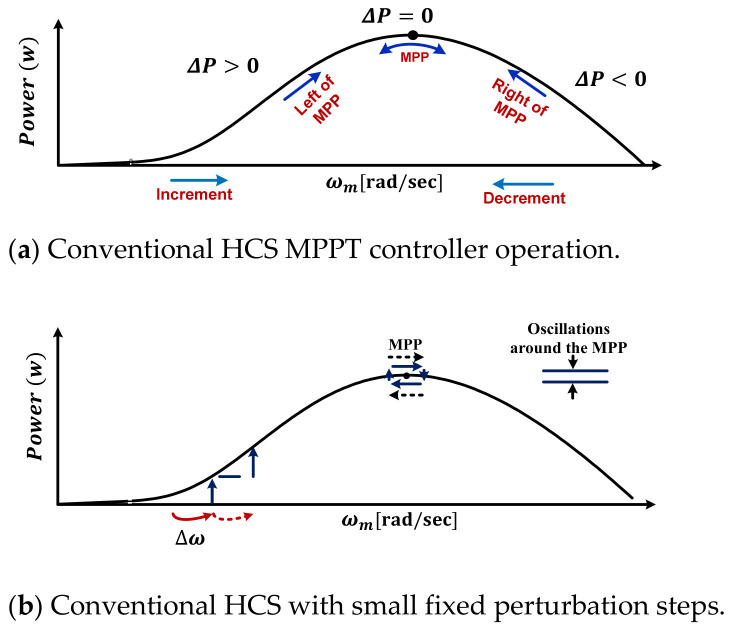

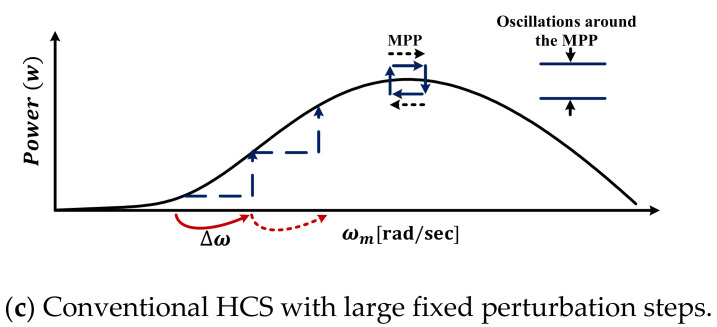
Conventional HCS MPPT strategy.

The conventional HCS technique is associated with variations in both harvested power and generator speed. If the slope $\frac{\mathrm{dP}}{d\omega }$ is larger than 0, the conventional technique increases the generator speed by a specified value in the same perturbation direction. Otherwise, the HCS reverses the perturbation direction, as portrayed in Figure 5a. Hence, the convergence speed and the oscillations around the MPP rely on the constant perturbation step size, which can be a small or large value, as revealed in Figure 5b and Figure 5c, respectively. When it uses the small step size, slight oscillations are gained with a slow convergence speed by which the power losses increase. Otherwise, the use of a large step size leads to the acceleration in the convergence speed; however, the oscillation levels are increased. Lastly, there are three drawbacks associated with the conventional HCS strategy during swift wind fluctuations, namely oscillations, convergence speed and MPP tracking failure with wrong perturbation direction. These issues lead to an increase in power losses and reduce the dynamic performance of the WT.

## 4. MRAC Strategy 

To achieve the best tracking effectiveness and WECS performance, Ȿ is used, instead of the fixed step in conventional or adaptive HCS techniques. Hence, the suggested technique depends mainly on Ȿ generated by the aid of the MRAC, which is considered as an online-updating step size without using prior knowledge of system parameters and memory requirements [54].

### 4.1. Description of the Suggested Methodology

**Figure 6:**
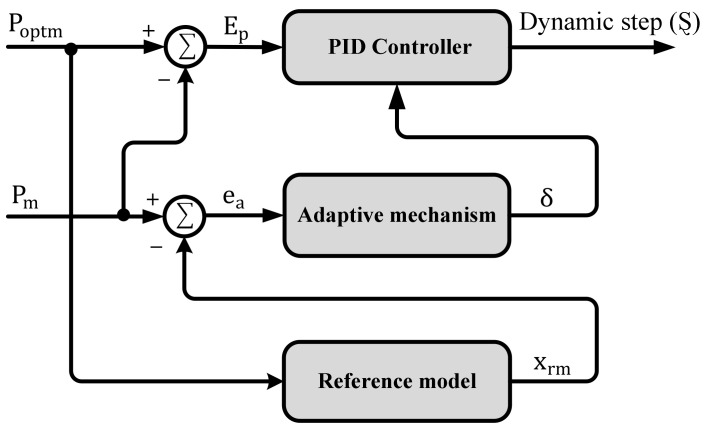
MRAC controller.

The use of a PID controller is one of the most widely applied control strategies in engineering applications [55]. This controller is commonly used because of its advantages such as high reliability and simplicity. However, there are several restrictions on its performance as a result of the uncertainty that exists in the WECSs. Therefore, the proposed control methodology uses an adaptive controller to avoid that. To cope with the rapid wind-speed fluctuations, MRAC is applied to adjust the PID controller parameters [56,57]. Hence, the PID controller parameters are varied continuously corresponding to the altering of the mechanical power $P_{m}$. Then, the PID output is used to adapt Ȿ for gathering the all-out wind power while dealing with several challenges such as the system uncertainty and the external disturbances. The configuration of the MRAC is based on three main subsystems, namely adaptive PID controller, reference model and adaptive mechanism [58,59], as depicted in Figure 6. 

Looking at the first part, the reference model is applied for capturing the desired behavior of the control system, which can be represented according to the second-order system as investigated in [60]. The transfer function of the second order ($G_{a}$) is designated as the reference model of the MRAC methodology, which exhibits the desired requirements of the control system (i.e., overshoot, settling time, rising time, and steady-state error). These requirements are satisfied when the system operates with an overshoot less than 5% and a settling time less than 2 s, providing an underdamped response. Here, the $G_{a}$ is represented as follows:

(20) $$G_{a}=\frac{x_{rm}}{P_{\mathrm{optm}}}=\frac{\alpha s+\omega_{n}^{2}}{s^{2}+2\zeta \omega_{n}s+\omega_{n}^{2}}$$

where ζ denotes damping ratio which equals ≅0.7, $\omega_{n}$ is the natural frequency ($\omega_{n}≅3\;\mathrm{rad}/s$) and α=0.

The mechanical power $P_{m}$ follows the reference model output $x_{rm}$ which is the desired reference trajectory from the second-order reference model. Whereas the WT has inner uncertainties and external disturbances, the MRAC should adapt the parameters to attain the desired response. On the other hand, the error $\;\left(e_{a}\right)$ between the output of the WT and the reference model must be zero; $e_{a}=0$, where

(21) $$e_{a}=P_{m}\text{-}x_{\mathrm{rm}}$$

Regarding the second part, the adaptive PID controller involves two parts: the adaptive mechanism and the traditional PID controller which has an output u.

(22) $$u=K_{P}\left[E_{P}\left(t\right)+\frac{1}{T_{i}}\overset{t}{\underset{0}{\int }}E_{P}\left(t\right).\mathrm{dt}+T_{d}.\frac{d(E_{p})}{\mathrm{dt}}\right]$$

(23) $$\mathrm{Where}\;\{\begin{array}{c} K_{i}=\frac{K_{P}}{T_{i}}\\ K_{d}=\frac{K_{P}}{T_{d}} \end{array}\;$$

$\;K_{P}$, $K_{I}$ and $K_{d}$ are the gains of proportional, integral and derivative controllers, respectively, and $T_{i}\;and\;T_{d}$ are the integral and derivative time constants, respectively. $E_{P}\left(t\right)$ is the controller error. Hence, the adaptive mechanism has two inputs, namely the error $e_{a}$ and the reference model output $x_{rm}$.

### 4.2. Adjustment of PID Parameters Using MRAC

The MRAC adjustment mechanism is an adaptive control rule constructed by the Massachusetts Institute of Technology (MIT). It is used to apply the MRAC strategy to all practical systems. The MIT adaptive control rule is considered as a gradient technique used to minimize the cost function j(θ) by adjusting the parameter θ [60]. In MRAC, the tracking error is measured as expressed in Equation (21). Hence, the cost function is the square of the model error function as follows:

(24) $$j\left(\theta \right)=\frac{1}{2}{\;e}_{a}^{2}$$

Therefore, it is logical to modify the adjustable parameter  θ in the direction of the negative gradient of j; that is [61],

(25) $$\frac{d\theta }{\mathrm{dt}}=-\gamma \;\frac{\partial J}{\partial \theta }=-{\gamma e}_{a}\;\frac{\partial e_{a}}{\partial \theta }$$

Here, the system sensitivity derivative $\partial e_{a}/\partial \theta$ illustrates the extent of error dependence on the adjustable parameter, θ.

By using MIT rules, the PID controller parameters become

(26) $$\frac{{\mathrm{dk}}_{p}}{\mathrm{dt}}=-\gamma_{p}\;\frac{\partial J}{\partial k_{p}}=-\gamma_{p}\left(\frac{\partial J}{\partial ɛ}\right)\left(\frac{\partial ɛ}{\partial P_{m}}\right)\left(\frac{\partial P_{m}}{\partial k_{p}}\right)$$

(27) $$\frac{{\mathrm{dk}}_{i}}{\mathrm{dt}}=-\gamma_{i}\;\frac{\partial J}{\partial k_{i}}=-\gamma_{i}\left(\frac{\partial J}{\partial ɛ}\right)\left(\frac{\partial ɛ}{\partial P_{m}}\right)\left(\frac{\partial P_{m}}{\partial k_{i}}\right)$$

(28) $$\frac{{\mathrm{dk}}_{d}}{\mathrm{dt}}=-\gamma_{d}\;\frac{\partial J}{\partial k_{d}}=-\gamma_{d}\left(\frac{\partial J}{\partial ɛ}\right)\left(\frac{\partial ɛ}{\partial P_{m}}\right)\left(\frac{\partial P_{m}}{\partial k_{d}}\right)$$

Finally, the output of the adaptive PID controller (Ȿ) can be formulated as

(29) Ȿ=u*δ

## 5. Proposed Adaptive HCS (AD-HCS) Technique

In order to optimize the generated power from the WECS, the rotor speed should be continuously regulated corresponding to the operating wind speed. Consequently, it is obligatory to proficiently operate at the MPP with fast response and small oscillations. 

To maximize the harvested wind power, a suitable step size must be applied despite continuous variations in the operating conditions. Looking deeply at the recent HCS techniques, it can be observed that most of them depend on the prespecified step size and system parameters. Furthermore, they increase the system complexity. To deal with these drawbacks, the MRAC continuously generates the required step size by detecting the mechanical power variations with respect to the optimal power without prior knowledge of system parameters and memory requirements. At that point, the proposed AD-HCS technique perturbs the rotor speed via simple HCS technique using Ȿ, which mainly depends on the output of the MRAC. Besides, MRAC optimizes the gains of the PID controller to specify the Ȿ well, as represented in Figure 2.

**Figure 7:**
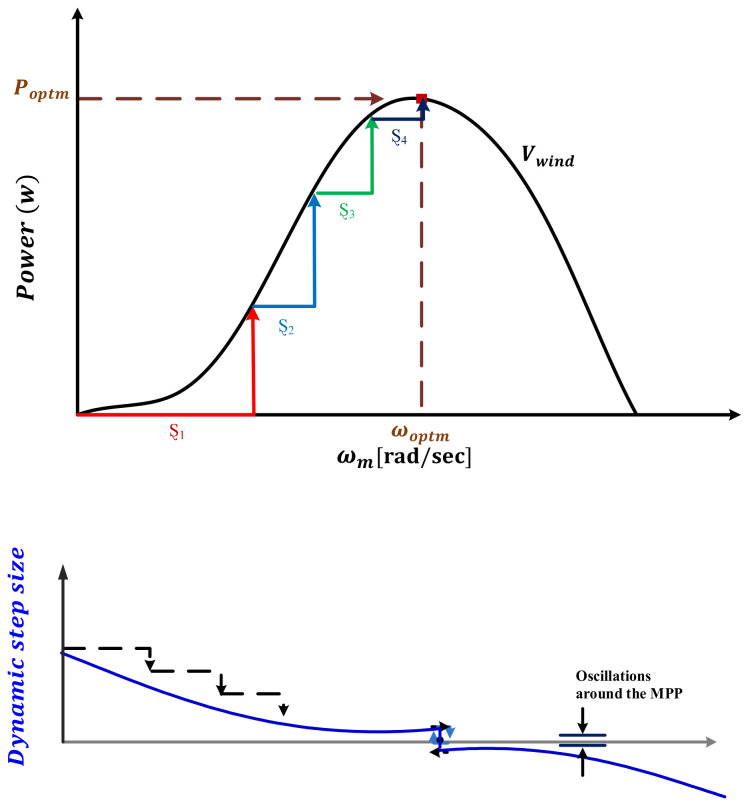
Proposed AD-HCS technique operation [62].

**Figure 8:**
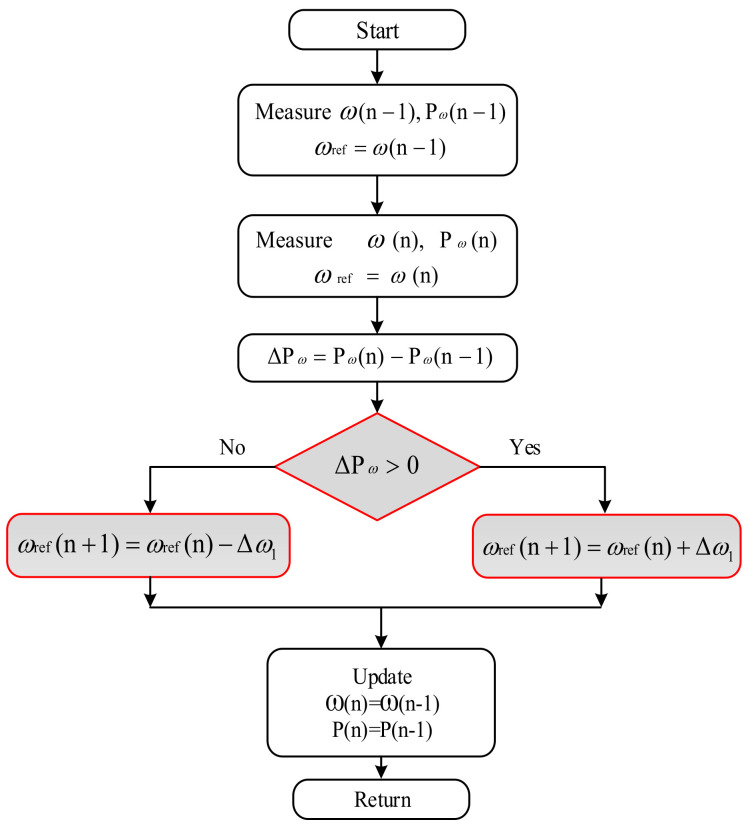
Flowchart of proposed AD-HCS technique.

Ȿ is related to the difference between the optimal power $\left(P_{optm}\right)$ and the mechanical power $\left(P_{m}\right).$ If the difference is large, Ȿ is large, as described in Figure 7, and vice versa. The complete flowchart of AD-HCS is portrayed in Figure 8. Hence, the suggested rotor speed value is specified as

(30) $$\Delta \omega_{1}=Ȿ\;*\;\Delta \omega$$

In summary, the proposed AD-HCS technique not only enhances the tracking strategy with ease of implementation but also provides a new adaptive method to optimize the step size without parameter sensitivity and the necessity of prior-knowledge constants, which is considered as a challenge when specifying them with wind speed variations. 

## 6. Simulation Results

The performance of the AD-HCS technique was certified under two different wind-speed profiles. In the first case, the wind-speed chart changes by steps with an average speed of 10 m/s. In the second case, the wind speed changes randomly with an average speed of 10 m/s and 20% turbulence. To show the high-performance capability of the proposed algorithm, its consequences are associated with the conventional and variable HCS (VS-HCS) techniques, which use specified step sizes. The conventional HCS technique was applied by using a large step size (LS-HCS) and a small step size (SS-HCS). On the other hand, the WECS overall efficiency was obtained over all the periods to notice the efficiency enhancement. All WECS parameters are presented in Appendix A (Table A1).

### 6.1. Step and Random Wind-Speed Change

**Figure 9:**
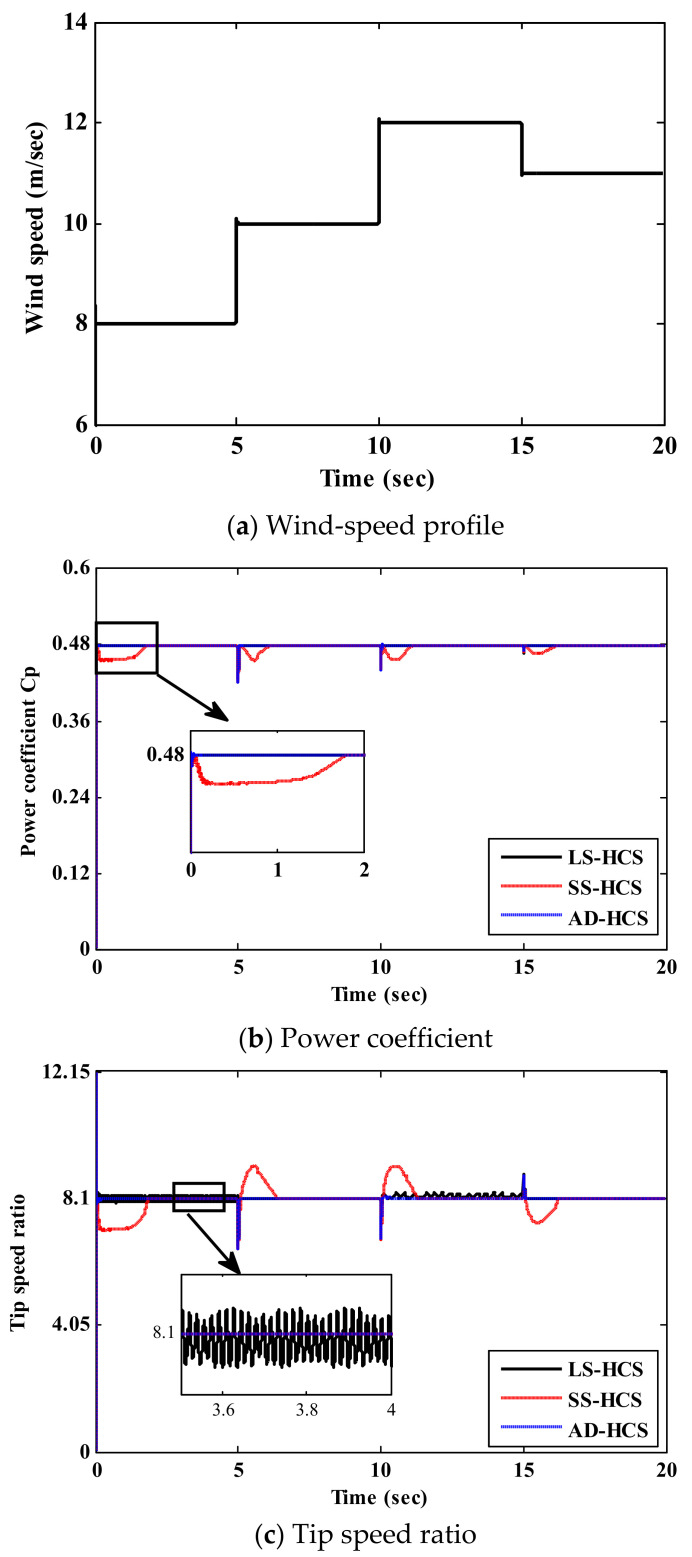

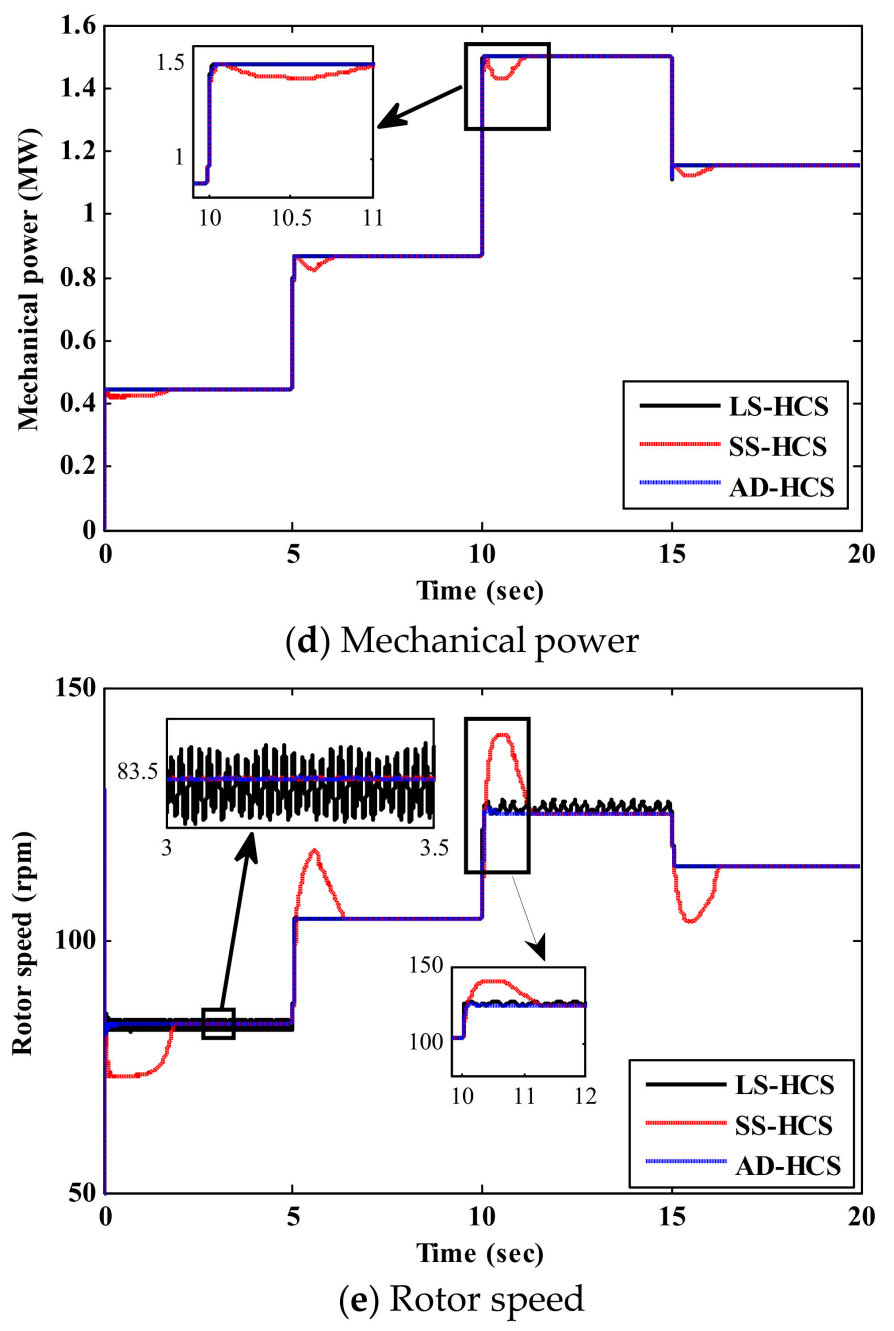
Simulation results under step-change wind speed.

**Figure 10:**
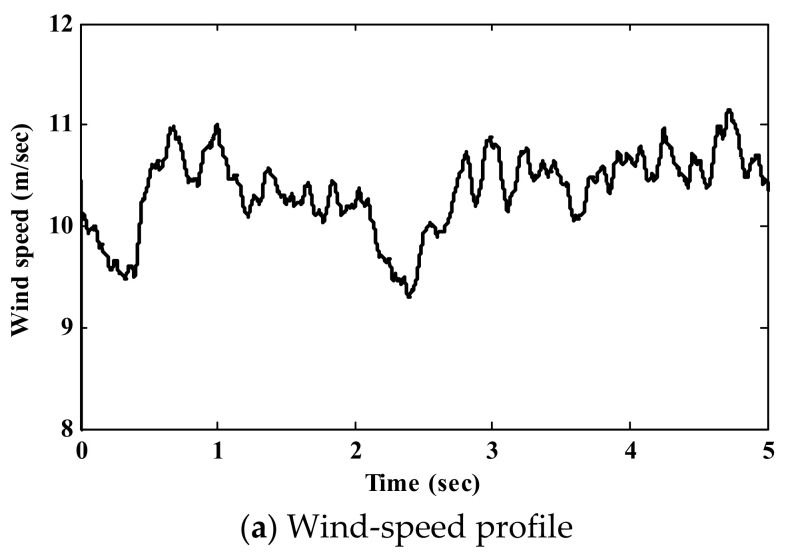

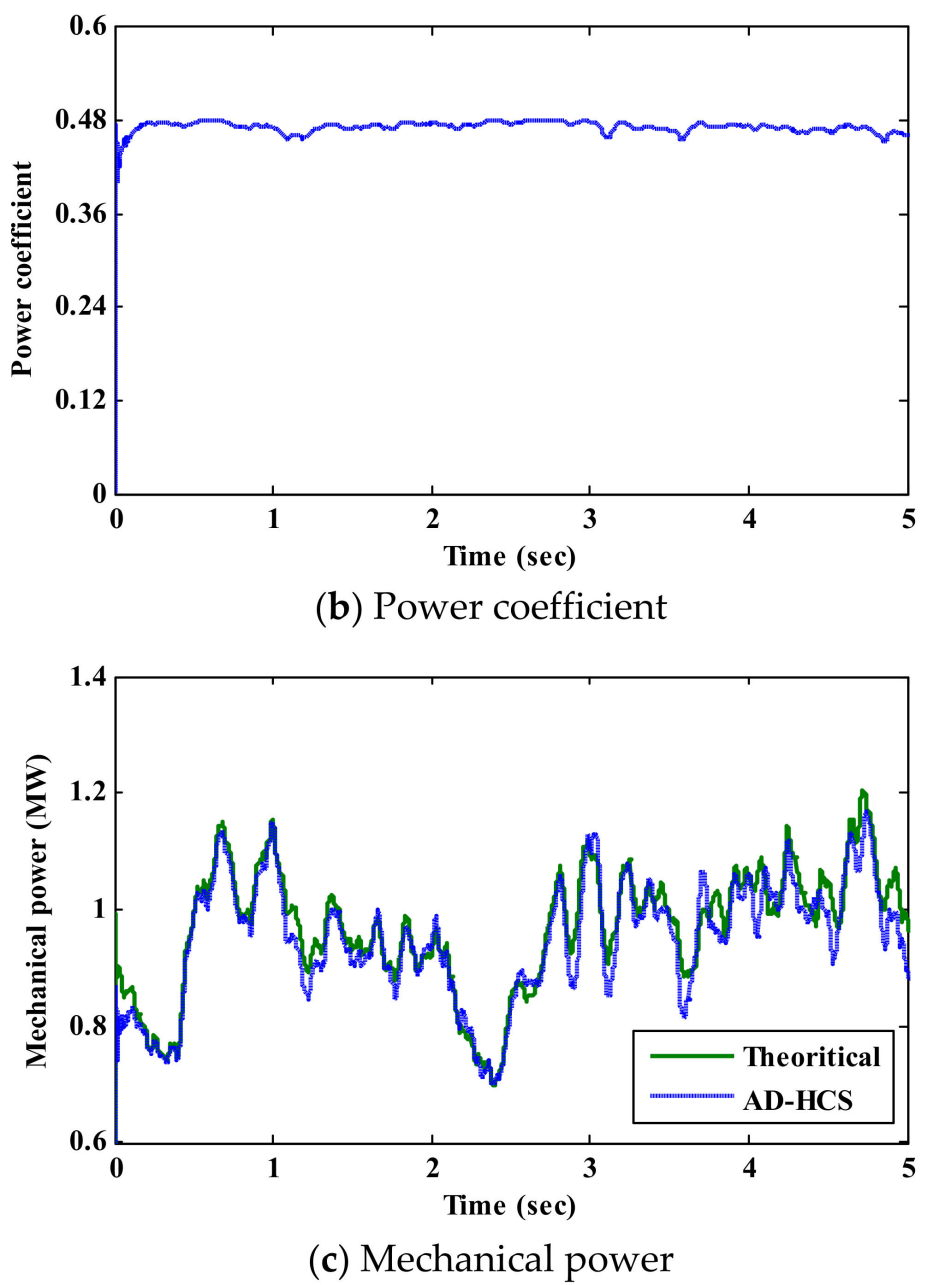
Simulation results under random wind speed.

The presented results of the suggested AD-HCS technique under the step variations in wind speed are portrayed in Figure 9. The wind-speed profile is shown in Figure 9a. The value of the power coefficient $C_{p}$, which specifies the ability of the WECS to extract the maximum power, is given in Figure 9b. It is clear that the AD-HCS technique tracks the optimal value $\left(C_{p}=0.48\right)$ more rapidly and with small oscillations compared to the conventional HCS techniques. Thus, the AD-HCS technique has a small settling time (42 ms). Here, the AD-HCS technique has a small oscillation (0.00942 rad/s), while the LS-HCS has large oscillations (2.5 rad/s). In steady-state conditions, the AD-HCS technique has low oscillation levels with high extracted power compared to the classical technique. As shown in Figure 9c, the proposed AD-HCS maintains the operating condition at the optimal value of λ (λ=8.1), which is more accurate than conventional HCS. This confirms that the proposed AD-HCS technique harvests the maximum power from the WECS during alternating wind speeds, as depicted in Figure 9d. The rotor speed follows the reference rate under wind speed variations, as portrayed in Figure 9e. Furthermore, it can be noticed that the proposed AD-HCS technique has a lower overshoot than both the LS-HCS and SS-HCS through wind speed instabilities. Regarding the simulation results under the step-change profile, the proposed AD-HCS technique exhibits efficient tracking performance compared to current HCS techniques. To study the performance of the proposed AD-HCS technique during rapid wind speed fluctuations, random wind speed was applied, as shown in Figure 10a. Figure 10b shows the resulting value of $C_{p}$ using the proposed AD-HCS. It can be observed that the proposed AD-HCS technique maintains the optimal value effectively with rapid response, which gives a good indication of power efficiency. Therefore, the output power from WT is maximum and tracks the optimal power, as illustrated in Figure 10c. 

### 6.2. Comparative Study

**Figure 11:**
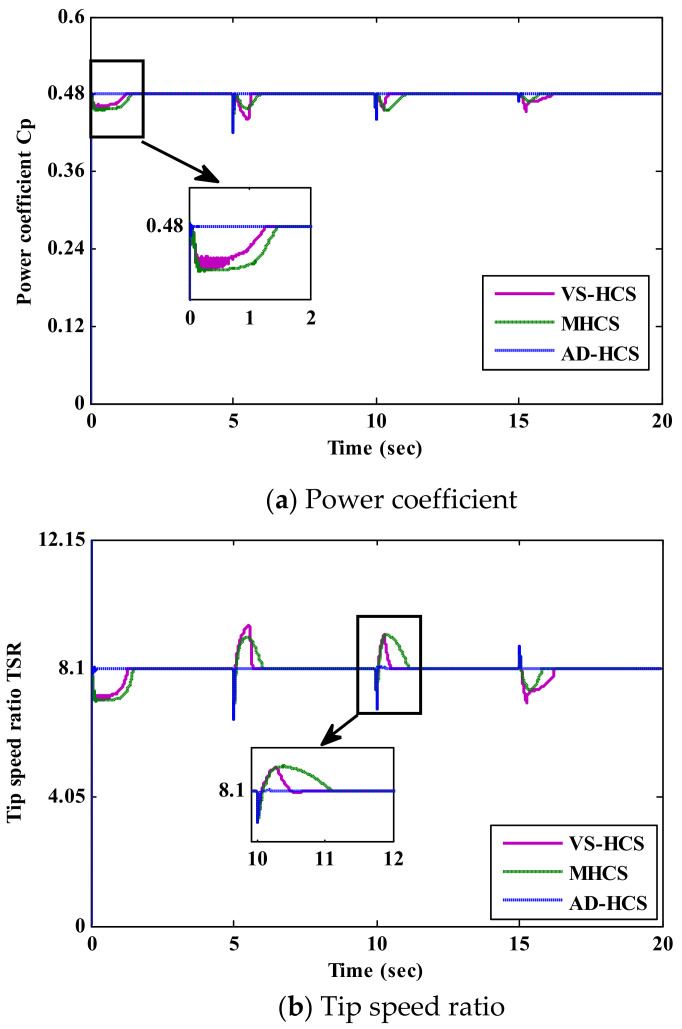

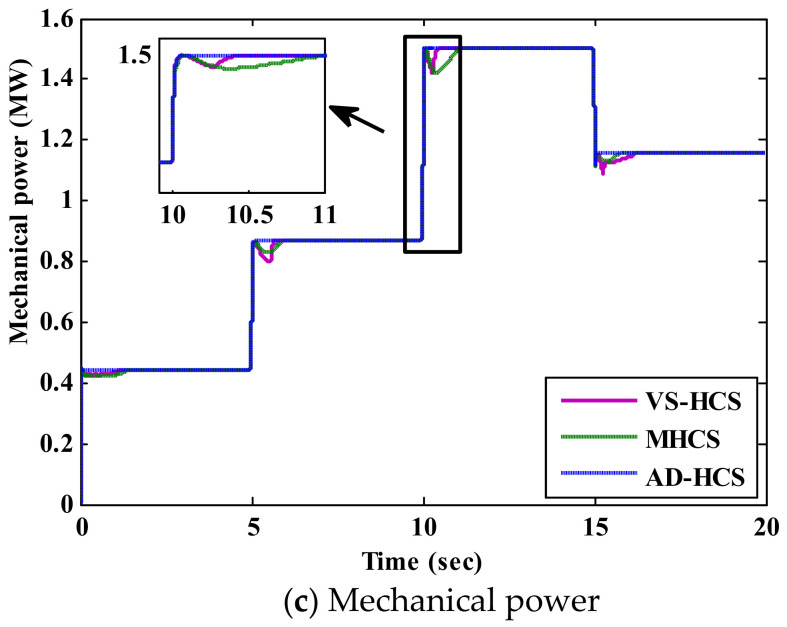
Comparative study of the proposed algorithm and current HCS algorithms.

Figure 11 presents a comparative study and performance assessment of the proposed AD-HCS technique and the recent variable and modified HCS (MHCS) techniques in terms of the power coefficient, TSR and extracted mechanical power. The presented figures depict the performance superiority of the proposed algorithm over the other current HCS algorithms. Firstly, the proposed AD-HCS technique operates with optimal power coefficient effectively with a settling time of 42 ms, whereas the settling times for VS-HCS and MHCS are 1200 and 1350 ms, respectively, as shown in Figure 11a. Thus, the proposed technique offers accurate tracking of MPP with a small settling time in comparison to other HCS techniques. As shown in Figure 11b, the proposed AD-HCS technique regulates the rotor speed with the optimal value of λ (λ = 8.1) in comparison to recent HCS techniques. Moreover, the proposed AD-HCS technique achieves the smallest peak-to-peak speed fluctuations (0.00942 rad/s), while the VS-HCS and MHCS techniques have the same large value of speed fluctuations (0.05 rad/s), which causes mechanical vibrations and bad effects on the overall drive-train. As a result of accurate MPP tracking, the proposed AD-HCS technique extracts the maximum power from the WECS with slight power fluctuations and a system efficiency of 89% at different wind speeds, as portrayed in Figure 11c. On the other hand, the system efficiencies of VS-HCS and MHCS techniques are 87% and 86.4%, respectively, due to the wrong tracking direction during perturbation of the rotor speed. Hence, the proposed AD-HCS technique is a vital solution to the several drawbacks of the existing HCS techniques, such as slow convergence speed, large speed ripples and wrong directionality under rapid wind speed variations. In addition, it reduces complexity in implementing the MPPT algorithm without requiring prior-stored initial constants and system parameters. Finally, it is clear that the proposed AD-HCS technique not only avoids the tracking loss, but also enhances the dynamic performance by accurately attaining the MPP with high performance, either in the transient or in the steady-state conditions. The numerical assessment of the proposed AD-HCS and the recent HCS techniques is presented in Table 2.

## 7. Conclusions

In order to harvest the maximum wind power, this paper suggests a new AD-HCS technique that overcomes the limitations of existing HCS techniques and improves their tracking performance and generated power quality based on a large-scale WECS. In spite of performance improvement of the existing HCS techniques, they suffer from using prespecified step size and requiring system parameters. Hence, the proposed AD-HCS technique adapts the step size during the mechanical power fluctuations using the MRAC. Firstly, the MRAC generates the required step size in order to continuously regulate the rotor speed instead of using the prior-knowledge step size. The choosing of the proper Ȿ depends mainly on optimizing the PID controller parameters by the MRAC to respond to continuous power variations. Then, the output step size is integrated into the simple tracking strategy. Regarding the simulation results, the proposed AD-HCS technique not only reduces the settling time but also enhances the overall WECS performance with low power oscillations around the MPP compared to the current HCS techniques. Hence, the proposed AD-HCS technique increases the WECS efficiency by 5% and 2% compared to the conventional and recent HCS algorithms, respectively. Finally, the proposed AD-HCS technique offers a simple tracking strategy and WECS performance enhancement without parameter sensitivity and the necessity of prior-knowledge constants during wind speed variations.

## Figures and Tables

**Table 1:** Literature review of recent HCS step size techniques.

Ref.	Details
[35]	This paper deals with the most common problems in HCS techniques such as speed–efficiency trade-off and wrong perturbation direction with fast fluctuations of wind speed. It follows the MPP contingent on an accurate value of $K_{opt}$, which is updated according to wind fluctuations. However, it is not possible to correctly track the MPP, as this technique necessitates measuring the wind speed to calculate $K_{opt.}$.
[36]	The relationship between the current and the square voltage of the DC-link is the basic idea for this technique, which enhances the system efficiency by 7.8%. On the other hand, the efficiency enhancement required offline training.
[37]	This tracking strategy via the sliding-mode controller has high efficiency under wind speed variations. Conversely, system modeling must be known.
[38]	The power lookup-table technique against speed characteristics was employed to obtain the MPP based on the field-oriented control. Furthermore, the unique $K_{opt}$ decreases tracking efficiency.
[39]	Authors investigated a modified HCS algorithm that considered the WT inertia. However, it requires a lookup table and powerful memory which depends on the WECS parameters
[40,41,42]	Intelligent MPPT techniques were created, mainly based on fuzzy logic control. However, these intelligent methods for extracting MPP require a considerable time interval (processing time) for hardware implementation.
[43]	The fixed perturbation steps were replaced by sinusoidal steps in the suggested HCS technique. This strategy is qualified for functioning efficiently only at fixed and slowly changing wind speeds. Moreover, it slows down the convergence speed.
[44]	A novel fast and efficient variable-step HCS technique was suggested; it divides the operating zone into modular operating zones by comparing a special synthesized ratio with the precise value. This method provides a vital solution; however, it uses a constant step size in each zone.
[45,46,47]	Recent P&O algorithms were investigated depending on the variation in step size with prior knowledge of system parameters and memory requirements.

**Table 2:** Comparison between the current HCS techniques and the proposed AD-HCS technique.

MPPT Techniques	Speed Ripple (P.P) rad/s	Δωrad/s	Settling Time (ms)	$\eta_{sys}$
**SS-HCS**	0.05	$\Delta \omega_{1}=$0.02	1900	84%
**LS-HCS**	2.5	$\Delta \omega_{1}=$0.2	60	--
**MHCS** [63]	0.05	$\Delta \omega_{1}=$0.1 $\Delta \omega_{2}=$0.02	1350	86.4%
**VS-HCS** [63]	0.05	Variable step size	1200	87%
**Proposed AD-HCS**	0.00942	Ȿ	42	89%

## Data Availability

Not applicable.

## Abbreviations

AD-HCS	Adaptive hill-climbing search
BTB	Back-to-back converter
DC	Direct current
DFIG	Double-fed induction generator
DPC	The direct power controller
GSC	Grid-side converter
HCS	Hill-climbing search
INC	Incremental conductance
IPC	Indirect power controller
LS-HCS	Large step size hill-climbing search
MIT	Massachusetts Institute of Technology
MRAC	Model reference adaptive control
MPP	Maximum power point
MPPT	Maximum power point tracking
ORB	Optimum relation-based
OT	Optimal torque
P&O	Perturb and observe
PID	Proportional integral derivative controller
PSF	Power signal feedback
RES	Renewable energy sources
RSC	Rotor-side converter
SS-HCS	Small step size hill-climbing search
TSR	Tip speed ratio
VS-HCS	Variable step size hill-climbing search
WECS	Wind energy conversion system
WT	Wind turbine

## Appendix A

**Table A1:** System parameters [64].

Specification of Wind Turbine	
**The coefficients C_1_ to C_6_**	$C_{1}=0.5176\;$,${\;C}_{2}=116$, $C_{3}=0.4$, $C_{4}=5$, $C_{5}=21$, $C_{6}=0.0068$
**Blade radius**	R=35.25 m
**Air density**	$\rho =1.225\;\mathrm{kg}/m^{3}$
**The optimal tip speed ratio**	$\lambda_{optm}=8.1$
**Maximum power coefficient**	$C_{p-optm}=0.48$
**DFIG parameters**	
**Rated power**	P=1.5 MW
**Pole pairs number**	$n_{p}=3$
**Stator resistance**	$R_{s}=0.023\;p.u$
**Stator inductance**	$L_{s}=0.18\;p.u$
**Moment of inertia**	H=0.685 s
**Mutual inductance**	M=2.9 p.u
**DC bus and gird parameters**	
**DC-link voltage**	$V_{\mathrm{dc}}=1150\;V$
**The capacitor of the DC-link**	C=0.01 F
**Grid voltage**	$V_{g}=575\;V$
**Grid frequency**	F=60 Hz
**Grid resistance**	$R_{g}=0.003\;\mathrm{pu}$
**Grid inductance**	$L_{g}=0.3\;\mathrm{pu}$
